# Correction: A Predictive Model of the Dynamics of Body Weight and Food Intake in Rats Submitted to Caloric Restrictions

**DOI:** 10.1371/journal.pone.0103664

**Published:** 2014-07-21

**Authors:** The PLOS ONE Staff

## Notice of Republication

This article was republished on July 1st, 2014, due to Figure 2, Figure 3, and Figure 4 being ordered incorrectly. Please download the PDF again to view the corrected article. The originally published, uncorrected article and the republished, corrected article are provided here for reference.

## Supporting Information

File S1 Originally published, uncorrected article
(PDF)

File S2 Republished, corrected article
(PDF)
