# Supplementary material for: Correction: A Predictive Model of the Dynamics of Body Weight and Food Intake in Rats Submitted to Caloric Restrictions
Source: PLoS One. 2014 Jul 21;9(7):e103664. doi: 10.1371/journal.pone.0103664 (PMC4105435; doi:10.1371/journal.pone.0103664)
Supplement: File S1 — Originally published, uncorrected article (PDF) [file pone.0103664.s001.pdf]

OPEN ACCESS Freely available online

PLOS ONE

A Predictive Model of the Dynamics of Body Weight and Food Intake in Rats Submitted to Caloric Restrictions

Marine Jacquier[1,2]*, Fabien Crauste[1,2], Christophe O. Soulage[3], Hédi A. Soula[3,4]

1 Université de Lyon, Université Lyon 1, CNRS UMR 5208, Institut Camille Jordan, Villeurbanne-Cedex, France, **2** Inria Team Dracula, Inria Center Grenoble Rhône-Alpes, Grenoble Rhône-Alpes, France, **3** CarMeN, INSERM U1060, Université de Lyon, INSA-Lyon, Univ. Lyon-1, Villeurbanne, France, **4** Project-Team Beagle, Inria Center Grenoble Rhône-Alpes, Grenoble Rhône-Alpes, France

Abstract

Dynamics of body weight and food intake can be studied by temporally perturbing food availability. This perturbation can be obtained by modifying the amount of available food over time while keeping the overall food quantity constant. To describe food intake dynamics, we developed a mathematical model that describes body weight, fat mass, fat-free mass, energy expenditure and food intake dynamics in rats. In addition, the model considers regulation of food intake by leptin, ghrelin and glucose. We tested our model on rats experiencing temporally variable food availability. Our model is able to predict body weight and food intake variations by taking into account energy expenditure dynamics based on a memory of the previous food intake. This model allowed us to estimate this memory lag to approximately 8 days. It also explains how important variations in food availability during periods longer than these 8 days can induce body weight gains.

Citation: Jacquier M, Crauste F, Soulage CO, Soula HA (2014) A Predictive Model of the Dynamics of Body Weight and Food Intake in Rats Submitted to Caloric Restrictions. PLoS ONE 9(6): e100073. doi:10.1371/journal.pone.0100073

Editor: Mihai Covasa, INRA, France

Received August 26, 2013; **Accepted** May 22, 2014; **Published** June 16, 2014

Copyright: © 2014 Jacquier et al. This is an open-access article distributed under the terms of the Creative Commons Attribution License, which permits unrestricted use, distribution, and reproduction in any medium, provided the original author and source are credited.

Funding: This work was funded by the authors' institutions: INSA Lyon, University Lyon 1, Inria and Inserm. The funders had no role in study design, data collection and analysis, decision to publish, or preparation of the manuscript.

Competing Interests: The authors have declared that no competing interests exist.

* Email: marine.jacquier@inria.fr

Introduction

Body weight regulation has become a major concern in our societies. A classical case of body weight dysregulation – obesity – is characterized by an excessive accumulation of white adipose tissue due to an energy imbalance between the energy derived from consumed food and the energy expended to maintain life [1–3]. Because obesity is recognized as an important health hazard [4], the causes of this imbalance have been extensively investigated in the past several years [5,6] with findings pointing out to peripheral as well as central mechanisms controlling food intake [7–9]. While feeding behavior – especially in human – can be difficult to assess, food intake behavior can be modulated by numerous factors, including but not restricted to nutrient signals – meal size and composition – and also orexigenic and anorexigenic hormones [10]. Among these hormones, ghrelin [11–13], cholecystokinin (CCK) [14], peptide YY [14], glucagon-like peptide-1 (GLP-1) [14] and leptin [15], have been identified as the main endocrine regulators of food intake. Anorexigenic gut peptides (CCK, GLP-1 and peptide YY) are produced in response to the presence of nutrients in the gastro-intestinal tract; their production is sensitive to changes in food composition such as an increase in fat content [16,17]. An increased level of ghrelin triggers feeding behavior and ghrelin production is decreased during the course of a meal [18]. On the other hand, leptin, a hormone secreted by adipose cells in proportion to white adipose tissue accretion, is known to trigger satiety [19].

All these hormones control the energy input. Yet, adaptation of the basal energy expenditure is another mechanism regulating food intake. It aims at reducing the difference between energy intake and the energy needed by the organism [20]. The latter can be modified by changes in activity and/or by adaptive thermogenesis (particularly in brown adipose tissue) [8,21,22]. In cases of overfeeding, thermogenesis is increased and ATP is wasted by completing futile cycles [23], while when underfeeding, energy expenditure is reduced to vital mechanisms [24]. This adaptation can prevent weight loss despite a reduced energy intake [22]. However it is not instantaneous and can be sustained, leading to important weight gains in individuals previously submitted to a strict diet. This is observed in humans and explains why body weight does not decrease linearly in time despite a constant reduction in caloric intake [25].

In normal conditions, these mechanisms should control weight variations. However some perturbations can destabilize this control. Our objective is to investigate mathematically whether variations in food availability could be the origin of such a destabilization.

Numerous mathematical or computational models describing metabolism regulation and body characteristics evolution exist in the literature. These models focus on different modelling scales, from cell to organism and from seconds to years [26]. Some models have been applied to animal subjects. Tam et al. [27] focused on physiological effects of leptin on energy homeostasis and food intake in mice. Guo and Hall [28,29] predicted dynamics of body weight and composition with respect to energy use in mice. Van Leeuwen et al. [30] studied the effect of food restriction on survival and body growth in mice. Other models have been applied to humans and were focused on energy use [31,32] or relationships between fat mass and fat-free mass [33,34]. These models were used to describe either normal conditions, overfeeding or starvation [35,36].

From the modelling point of view, feeding behavior and hunger have been relatively ignored. Although some results on the feeding dynamics correlate with body mass index [37], no other modelling work has ever studied the impact of food availability dynamics on the feeding behavior and body weight regulation.

In this paper, we propose a mathematical model of body weight dynamics (divided in fat mass and fat-free mass), taking into account hunger, defined throughout this manuscript as the amount of food needed by the organism, leptin, ghrelin and glucose variations. Food intake is assumed to be regulated by the available amount of food and by hunger. As we focus on the influence of available and consumed food, we have chosen to consider only leptin (as an indicator of fat storage), ghrelin (representative of the volume of food intake) and glucose (proportional to the energy content of the diet) amongst all the factors influencing food intake. Unlike other published models, this system includes a memory of past food intake to model the adaptation of energy expenditure to caloric restrictions.

To challenge the model and find relevant parameter values, we conducted a simple feeding experiment on rats. One group received *Ad libitum* food. The time course of the available food for the three other groups was modified with three different frequencies, while maintaining an isocaloric diet during the entire experiment. We show that low frequency perturbations are very likely to induce weight gains and that our model is able to predict this feature.

Materials and Methods

Experimental Procedures

Animal care. Animal experiments were performed under the authorization n°69-266-0501 (INSA-Lyon, DDPP-DSV, Direction Départementale de la Protection des Populations - Services Vétérinaires du Rhône), according to the guidelines laid down by the French Ministère de l'Agriculture (n° 87–848) and the E.U. Council Directive for the Care and Use of Laboratory Animals of November 24th, 1986 (86/609/EEC). COS (n° 69266257) holds a special license to experiment on living vertebrates issued by the French Ministry of Agriculture and Veterinary Service Department.

Thirty twelve-week-old Wistar rats were purchased from Janvier SA (Le Genest-Saint-Isle, France) and housed in an air-conditioned room at $24\pm1^{\circ}$C with a LD (light/dark) 12:12 cycle (light on at 6:30 am) with free access to food (2016C, 12.6 kJ/g, 66% carbohydrates, 12% fat, 22% proteins, Harlan, Gannat, France) and water.

Rats were randomly separated into 5 groups (D0, AL, H0, H1 and H4) of 6 individuals (no significant difference of initial body weight was found between these groups: *p-value* = 0.26). Each rat was identified and housed individually throughout the protocol.

The group D0 was sacrificed on the first day of the experiment (as described below), so the initial biometric data of the rats are available, including body weight, body length, white adipose tissue mass, brown adipose tissue mass, muscles and organs weights (see Table 1). At the end of the experiment (i.e. 8 weeks) the other rats were sacrificed to obtain the same data. Blood samples were collected at the same time for further analyses. The total body lipid content can easily and accurately be predicted from the gravimetric determination of the retroperitoneal fat deposits [38]. Thus the retroperitoneal fat pads weights (rWAT) were used to estimate the total body lipid content (L in grams), using the formula $L = 7.96\text{rWAT} + 3.13$ [38].

Rats from groups AL, H0, H1 and H4 were individually housed and received chow diet for 8 weeks in different quantities each day (see Fig. 1). The control group AL received *Ad libitum* food (approximately 25 g per rat per day). *Ad libitum* food also corresponds to the diet before the beginning of the experiment in each group. The other groups (H0, H1, H4) were submitted to a restriction in caloric availability corresponding to 80% of *Ad libitum* diet. This reduction should theoretically avoid leftovers, as this amount is below normal consumption, in order to have a better control on food intake.

The pattern of food distribution was not the same for these three groups. Group H0 received the same amount of food every day for 8 weeks. For the H1 group the food was randomly allocated for each week of the experiment. Group H4 was submitted to an important restriction for 4 weeks followed by an excess of food for the remaining 4 weeks. The amount of food given each day is reported in Fig. 1. The remaining food was measured and removed each day to determine the food really consumed (see Fig. 2 B). Great care was taken to ensure that most of the food was either eaten or removed and not wasted. In a preliminary experiment, we determined that food spillage only accounts for $6.9\pm1{,}0\%$ of the total food intake. Therefore it was considered to be negligible.

During the experiment, the beginning of the week (the day the food availability was changed) was set on Tuesday and rats were weighted every Friday morning. This protocol tends to minimize and to separate the effects of stress due to changes in food availability and stress due to weighting.

Sacrifice, blood and tissue collection. Animals were deeply anesthetized with sodium pentobarbital (60 mg/kg ip), blood ($\sim$5 mL) was collected through puncture of vena cava on heparinized syringe and centrifuged 2 min at 8000 g. Plasma samples were snap frozen in liquid nitrogen and stored at -80°C until analysis. Liver, heart, kidneys, gastrocnemius muscles, epididymal, retroperitoneal and subcutaneous inguinal white adipose tissue (WAT) were dissected out according to anatomical landmarks, weighed to the nearest milligram, snap frozen in liquid nitrogen and stored at -80°C. Total WAT mass was calculated as the sum of the mass of epididymal, retroperitoneal and subcutaneous inguinal WAT deposits.

Individual data is freely available upon request.

Biochemical analysis. Plasma ghrelin and leptin assays were performed using immunoassays (acylated rat/mouse ghrelin #A05117 and rat/mouse leptin EIA #A05176, Cayman, SpiBio, Montigny le Bretonneux, France) according to the manufacturer's recommendations. The detection limit and intra-assay coefficient of variation for ghrelin were 0.2 pg.mL^{-1} and 11%, respectively. The detection limit and intra-assay coefficient of variation for leptin were 50 pg.mL^{-1} and 4%, respectively. Blood glucose was measured using an automatic glucose monitor (Optium Xceed, Abbott, Rungis, France). All assays were performed at least in duplicate (see Table 2).

Individual data is freely available upon request.

Mathematical Model

In this section, the mathematical model is described (See Table 3 for a description of all variables and Fig. 3 for a schematic representation of the system). This model focuses on fat mass and fat-free mass evolutions regulated by hunger and available food. Hunger is defined as the amount of food the system would consume were there no constraint on food availability. There exist multiple factors influencing food intake [7–10,14,39], yet we focus on 3 of them: leptin, ghrelin and glucose (which is highly correlated with insulin) as they regulate hunger at different time scales [8].

Table 1. Biometric data.

	D0	AL	H0	H1	H4
Body weight (g)	333.8±15.9	493±46.7	410.5±24.1	404.8±40.5	444.5±16.4
Body length (cm)	22.9±0.53	26.4±0.51	25.4±0.66	25.1±0.75	26±0.67
rWAT (g)	4.24±1.49	9.77±2.30	8.82±3.80	7.63±1.56	9.58±2.61
Total WAT (g)	17±4.6	33.3±9.4	29.2±9.7	28.2±4.7	32.9±5.4
iBAT (mg)	287±39	425±199	365±71	367±64	390±153
Kidneys (g)	2.37±0.59	3.11±0.22	2.56±0.04	2.69±0.35	2.70±0.34
Heart (g)	0.96±0.09	1.40±0.13	1.16±0.05	1.24±0.12	1.19±1.13
Soleus (g)	235±48	248±37	172±33	203±24	183±45
EDL (g)	110±37	165±44	193±18	188±12	192±51

Each column gives biometric data for the 5 groups: for all groups except D0 (group sacrificed on the first day of the experiment), data have been obtained at the end of the experiments.
WAT = White Adipose Tissue, rWAT = retroperitoneal White Adipose Tissue, iBAT = interscapular Brown Adipose Tissue, EDL = Extensor digitorum longus.
doi:10.1371/journal.pone.0100073.t001

Fat mass (S, in grams) and fat-free mass (W, in grams) are assumed to be produced depending on the instantaneous difference (Δ_E) between energy intake and energy expenditure. To model this phenomenon we adapted the equations in [28,29] previously developed for a mouse model and we used the same notations: ρ_S and ρ_W denote the energy densities for fat mass and fat-free mass respectively (kJ.g^{-1}) and Δ_E the instantaneous difference of energy (kJ.min^{-1}). Evolutions of S and W are given by:

$$\frac{dS}{dt} = \frac{\Delta_E}{\rho_W x + \rho_S}, \qquad (1)$$

$$\frac{dW}{dt} = \frac{\Delta_E x}{\rho_W x + \rho_S}, \qquad (2)$$

where $x \equiv dW/dS = \zeta + \psi.\exp(\kappa.S)$ [28,29].

Energy intake (EI) is supposed to be a function of the caloric content of the diet. This food consumption per minute, denoted by $c(a,h)$ (kJ.min^{-1}), is assumed to be a function of hunger h (kJ) and available food a (kJ). We assume $c(a,h)$ is equal to the minimum of a and h per unit of time. Hunger was defined as the amount of food needed by the system (see above). Hence, food consumption is either equal to hunger, when enough food is available or to the available food a.

Several formulae describe energy expenditure [34,40], using linear dependencies on body weight, fat mass and fat-free mass. In the current model, the energy expenditure (EE) is assumed to be a function of the caloric content of the body (expressed as a function of fat mass and fat-free mass) with a rate of energy expenditure R. The result is the amount of Joules lost per minute. We then define the energy balance Δ_E as:

$$\Delta_E = EI - EE = c(a,h) - R(\rho_W W + \rho_S S + \xi),$$

where $EI = c(a,h)$ and $EE = R(\rho_W W + \rho_S S + \xi)$.

One can note that fat-free mass has a negative feedback on itself and that fat-mass may have either a positive or negative feedback on itself, depending on the value of Δ_E. Fat mass can have a positive feedback on fat-free mass, via x (see equation (2)), since creation of fat mass leads to the creation of lean mass [33].

The evolution of the amount of available food a (in kJ) depends on the input of food in the system f (usually a given amount each day) and the consumption c. The available food a satisfies

$$\frac{da}{dt} = f(t) - c(a,h). \qquad (3)$$

In order to describe variations in appetite, the model should take into account the evolution of factors influencing hunger. As previously mentioned, we focus on leptin, glucose and ghrelin concentration. The total plasma leptin l (in ng) is assumed to be produced proportionally to the fat mass [27],

$$\frac{dl}{dt} = \gamma_2 S - \gamma_1 l. \qquad (4)$$

Group	Week 1	Week 2	Week 3	Week 4	Week 5	Week 6	Week 7	Week 8
AL	*Ad libitum*							
H0	20 g	20 g	20 g	20 g	20 g	20 g	20 g	20 g
H1	15 g	25 g	10 g	10 g	30 g	35 g	5 g	30 g
H4	10 g	10 g	10 g	10 g	30 g	30 g	30 g	30 g

Figure 1. Daily available food per rat in each group. Changes of quantities occur each week, except for group AL which was not submitted to caloric restriction. At the end of the 8-weeks experiment, each rat in groups H0, H1 and H4 will have received 1120 g of food. Consumed food is not always equal to this amount but is recorded every day.
doi:10.1371/journal.pone.0100073.g001

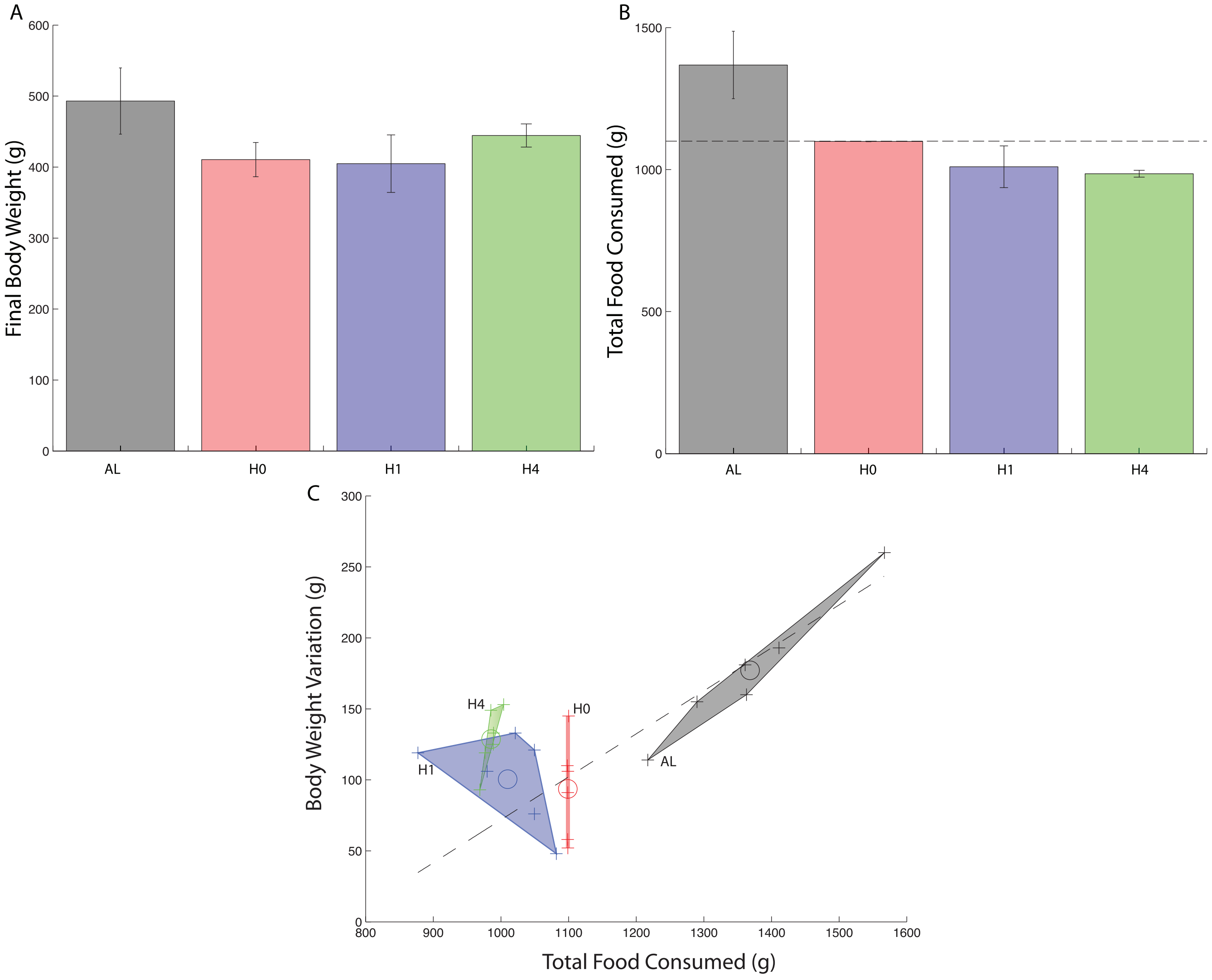

Figure 2. Body weight and food intake evolution. A) Temporal evolution of body weights (in grams) for each group (mean ± sd): AL (black), H0 (red), H1(blue) and H4 (green). A small offset has been added to the time points to ease the reading. **B)** Evolution of consumed food (straight lines, mean ± sd) weekly by each rat (in each group: AL (black), H0 (red), H1(blue) and H4 (green)) compared to the available food (dashed lines). Group H0 consumed all its available food for the duration of the experiment while groups H1 and H4 had leftovers.
doi:10.1371/journal.pone.0100073.g002

Total glucose u (g) and ghrelin concentration e (pg.mL^{-1}) in plasma depend on the diet composition [18]. The glucose level increases with food intake as follows:

$$\frac{du}{dt} = \mu_1 c(a,h) - \mu_2 u. \quad (5)$$

Table 2. Plasma hormones and glucose assays.

	D0	AL	H0	H1	H4
Ghrelin (pg.mL^{-1})	nd	43.30 ± 17.75	25.66 ± 14.65	78.48 ± 97.96	18.01 ± 9.88
Leptin (ng.mL^{-1})	4.34 ± 1.78	7.53 ± 2.72	3.60 ± 1.66	5.19 ± 1.66	7.07 ± 3.66
Glucose (mg.dL^{-1})	140.7 ± 11.6	148.7 ± 24.1	132.2 ± 8.9	121.4 ± 14.8	144.3 ± 21.2

Ghrelin, leptin and glucose concentrations in plasma in the control group and at the end of the experiment for groups AL, H0, H1 and H4 (mean ± sd, nd: not determined).
doi:10.1371/journal.pone.0100073.t002

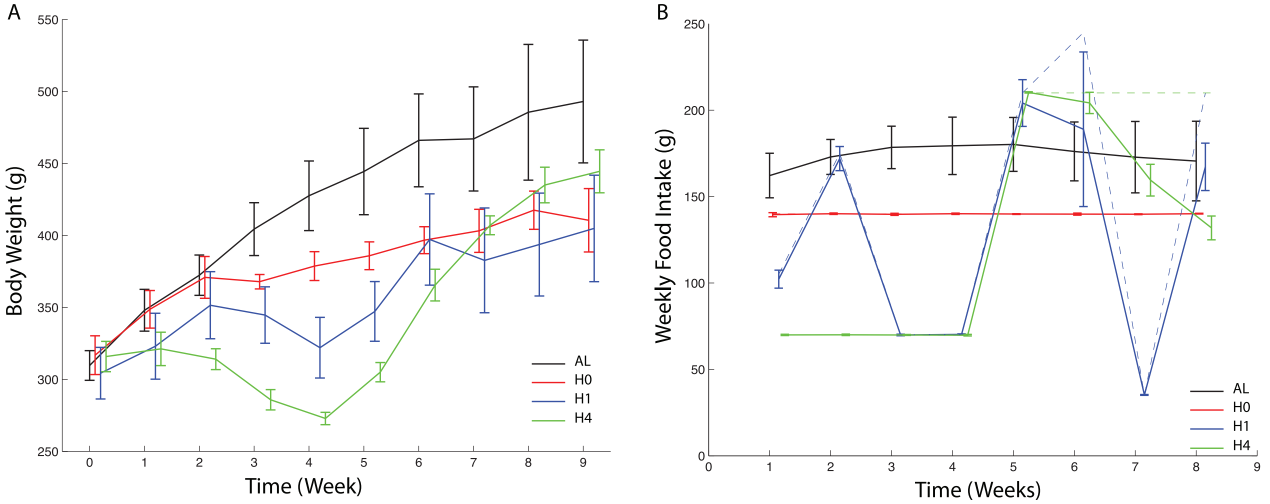

Figure 3. Schematic representation of the model. Positive influences are represented by straight lines with arrows and negative influences by bar-headed lines. Relations whose effect can vary in time are represented by dashed lines with a dot at the end.
doi:10.1371/journal.pone.0100073.g003

Ghrelin production is inhibited in the presence of food in the stomach [41],

$$\frac{de}{dt}=\frac{v_2}{1+v_1 c(a,h)}-v_3 e. \tag{6}$$

The hunger h is regulated in the central nervous system, integrating signals from the rest of the body via circulating hormones [7,8]. Regulation of hunger is a complex system. The amount of circulating leptin as an indicator of body adiposity leads to a decrease in hunger [8], so we assume hunger decreases when leptin increases. The ghrelin concentration decreases when the stomach is full and the hunger follows the same variations [12] so we assume hunger increases when ghrelin increases. The effect of leptin and ghrelin is opposite, though they both have an action in the arcuate nucleus [18]. The hunger h is also supposed to be a decreasing function of glucose level u [42]. The hunger h was defined as the amount of Joules required by the system at any time, so the evolution of h is given by:

Table 3. Model variables.

Name	Symbol	Unit
food available	a	kJ
hunger	h	kJ
plasma ghrelin	e	pg.mL^{-1}
plasma glucose	u	g
plasma leptin	l	ng
fat mass	S	g
fat-free mass	W	g
rate of energy expenditure	R	min^{-1}

Variables of the model with associated units and symbols.
doi:10.1371/journal.pone.0100073.t003

$$\frac{dh}{dt}=\frac{\alpha_1 e}{1+\alpha_2 l}-\beta(\alpha_3+u)h. \tag{7}$$

System (1)–(7) takes regulations at short and long time scales into account. Variables directly linked to daily food intake such as ghrelin concentration and glucose level have an influence on a daily basis whereas leptin has an influence on a longer time scale.

Adaptation of Energy Expenditure

The previously described model is well adapted when food is available *Ad libitum*. As the food consumed is always equal to hunger, the organism does not need to change relatively to environmental conditions and its rate of energy expenditure R is therefore constant. In the case of caloric restrictions, energy expenditure is lowered to maintain the energy balance [43]. To take this phenomenon into account, we assume that the rate of energy expenditure R depends on the food consumed, with a memory effect.

The rate of energy expenditure R is known to adapt to the past food intake $c(a,h)$ [24]. As the food is supposed to be available on a daily basis, the mean food intake in the last τ days is compared to the mean food intake in the last τ' days (with $\tau'>\tau$) to define the value of R. The "reference" food (food consumed between times $t-\tau'$ and t) is slowly modified accordingly, so R is constant if the food intake doesn't change for at least τ' days. When food intake varies on short periods of time, the rate of energy expenditure R is progressively modified to reduce the difference between these mean food intakes, with a rate of adaptation equal to ϵ, as follows:

$$\frac{dR}{dt}=\epsilon\left(\frac{1}{\tau}\int_{t-\tau}^{t}c(a(v),h(v))dv-\frac{1}{\tau'}\int_{t-\tau'}^{t}c(a(v),h(v))dv\right). \tag{8}$$

This equation needs an initial condition R_0 which corresponds to the value of the rate of energy expenditure with a constant food intake equal to hunger (*Ad libitum* case).

Table 4. Model parameters.

Parameter	Value	Unit	
ν_1	1.52	min.kJ^{-1}	experiments
ν_2	0.4025	pg.mL^{-1}.min^{-1}	experiments
ν_3	0.007	min^{-1}	[46]
μ_1	0.039	g.kJ^{-1}	diet composition
μ_2	0.007	min^{-1}	[47]
γ_1	0.074	min^{-1}	[48]
γ_2	0.126	ng.g^{-1}.min^{-1}	[48] and experiments
ρ_W	7.5	kJ.g^{-1}	[29]
ρ_S	39.3	kJ.g^{-1}	[29]
ξ	1964.4	kJ	fit step 1
ζ	2.2	–	fit step 1
ψ	1.6 $\times 10^{-9}$	–	fit step 1
κ	0.269	g^{-1}	fit step 1
R_0	2.525 $\times 10^{-5}$	min^{-1}	fit step 1
α_1	4.02 $\times 10^{-8}$	mL.kJ.min^{-1}.pg^{-1}	fit step 2
α_2	1.66 $\times 10^{-4}$	ng^{-1}	fit step 2
α_3	5.03 $\times 10^{-4}$	g	fit step 2
β	5.99 $\times 10^{-6}$	min^{-1}.g^{-1}	fit step 2
ϵ	9.05 $\times 10^{-9}$	kJ^{-1}	fit step 3
τ	1	day	fit step 3
τ'	8	day	fit step 3

Values of the parameters used in the model and associated units. When the parameter is taken from the literature, the corresponding reference is indicated.
doi:10.1371/journal.pone.0100073.t004

Other factors influence energy expenditure [20,39] such as plasma leptin, environment and aging [43,44]. Nevertheless this model focuses only on the effect of caloric variations as it is the easiest parameter to measure and manipulate experimentally.

Parameter Estimation

System (1)–(8) use 21 parameters whose values are essential to the relevance of the simulation results. Amongst these, the 4 parameters of hormone production and degradation are taken from the literature (see Table 4 for a summary of units and origins of parameters of the model). Food-relative parameters depend on the composition of the chow diet.

To estimate the 12 remaining parameters, we used the final fat mass and the evolution of body weight of each individual rat from groups AL and H1. Parameter values were obtained by minimizing the residual sum of squares (RSS) of observed data compared to simulation results using the Nelder-Mead algorithm [45]. We then used these parameter values to test the predictive capacity of our model against data from groups H0 and H4.

In the AL case, *Ad libitum* food implies that food intake c is always equal to h and the rate of energy expenditure R is constant. Consequently, energy expenditure only depends on fat mass S and fat-free mass W. We have access to the experimentally consumed food, so we use this value to explicitly determine the evolution of h. Hence equations (1) and (2) are decoupled from the other equations and we consider them as an independent subsystem.

In a first step, applying the minimization algorithm to this subsystem leads to an estimation of the 4 parameter values relative to equations (1) and (2). Then, in a second step, we estimate the remaining parameter values using equations (1), (2), (4), (5), (6) and (7) and the previously determined parameters. To estimate the parameters relative to h in equation (7) we once again use data from group AL. However, this time, c was determined by the values of a corresponding to *Ad libitum* food and h is given by (7). As R is supposed to be constant over group AL, the system used here was composed by all equations except (8). Finally, those parameters relative to the rate of energy expenditure R, were estimated in a third step, using experimental data from hypoca-

Table 5. Approximate bayesian computation of parameters.

	ϵ (kJ^{-1})	τ (day)	τ' (day)
mean	1.01×10^{-8}	1.3	8.4
standard deviation	0.74×10^{-8}	0.9	6.1

Mean and standard deviation of selected runs of the ABC (RSS $<$ 1.3 RSS$_{opti}$) for parameters relative to the memory of the system (ϵ, τ and τ'). Mean values are close to parameter values estimated with the optimisation process but with an important standard deviation around these values.
doi:10.1371/journal.pone.0100073.t005

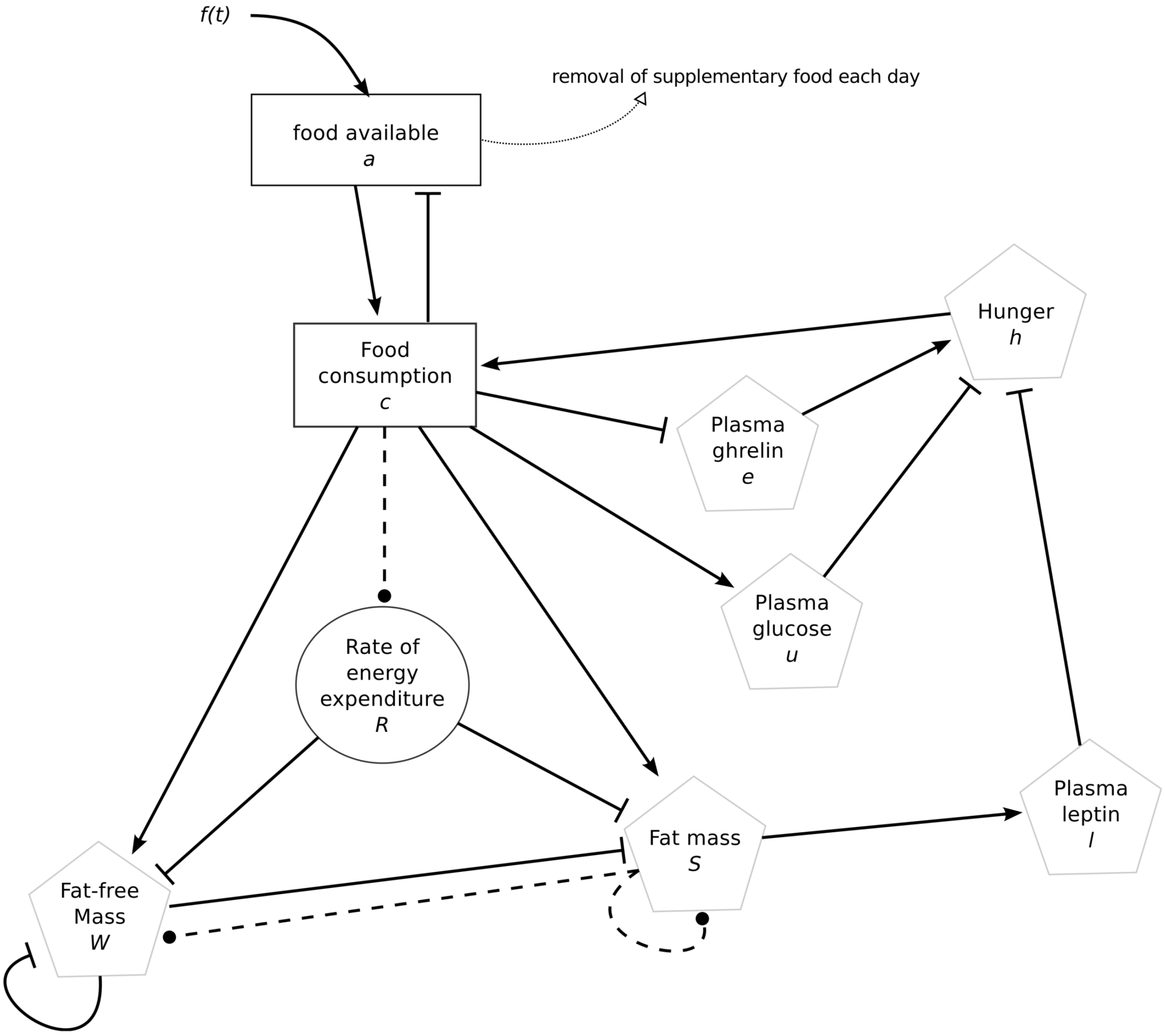

Figure 4. Final experimental body weight and food intake. A) Final body weights at the end of the experiment for all groups. All hypocaloric groups (H0, H1, H4) are significantly different from group AL. Within hypocaloric groups, H4 is significantly different from H0. **B)** Total amount of food consumed in grams at the end of experiment (8 Weeks) for all groups (mean $\pm$ standard deviation). All hypocaloric groups (H0, H1, H4) consumed significantly less food than group AL. Within hypocaloric groups, H4 is significantly different from H0 and H1. Dashed line is the amount of the total food that was available for the hypocaloric group. **C)** Variation of body weight from the start to the end of the experiment versus the total amount of food consumed. Crosses indicate individual points, open circles are the averages and the whole group is described by its convex hull. The dashed line is the linear regression for the group AL ($p = 0.005$ and $R^2 = 0.88$). The slope is 0.3, indicating that the weight gain is equal to 30% of the weight of the consumed food.
doi:10.1371/journal.pone.0100073.g004

loric group H1. We then used the whole system of equations and parameter values previously estimated for AL.

Parameter estimation is detailed as follows:

Step 1. Only equations (1) and (2) are used. The input of the system is the experimentally determined consumed food c for group AL. We assume R is equal to R_0 when Ad libitum food is available. As we have a value for c, it is not necessary to describe the variations of hunger and hormones so the only dynamical variables of the subsystem are S and W. The RSS between outputs of the model (predicted body weight and fat mass) and experimental data (body weight and fat mass) is minimized for each individual rat from group AL. This leads to an estimation of the parameters ξ, κ, ψ, ζ and the basal rate of energy expenditure $R = R_0$.

Step 2. Equations (1), (2), (4), (5), (6) and (7) are used with experimental data from group AL. Parameter values determined at step 1 are used at this step. The rate of energy expenditure R is still supposed to be constant as the food is Ad libitum, with $R = R_0$ determined in the previous step. In this step, $c(a,h)$ is supposed to be equal to h as a is always higher than h (to take unlimited food into account). This leads to an estimation of the parameter values relative to the hunger h: α_1, α_2, α_3 and β.

Step 3. For the last step, the whole system is used. Both the pattern of food availability and experimental data from group H1 are used, with five initial days of Ad libitum food to be consistent with the experiment. Parameters determined at steps 1 and 2 are used. As the H1 rats are supposed to adapt to the reduced and varying amount of food available, this allows to estimate the parameters associated with energy expenditure variations: ϵ, τ and τ' in

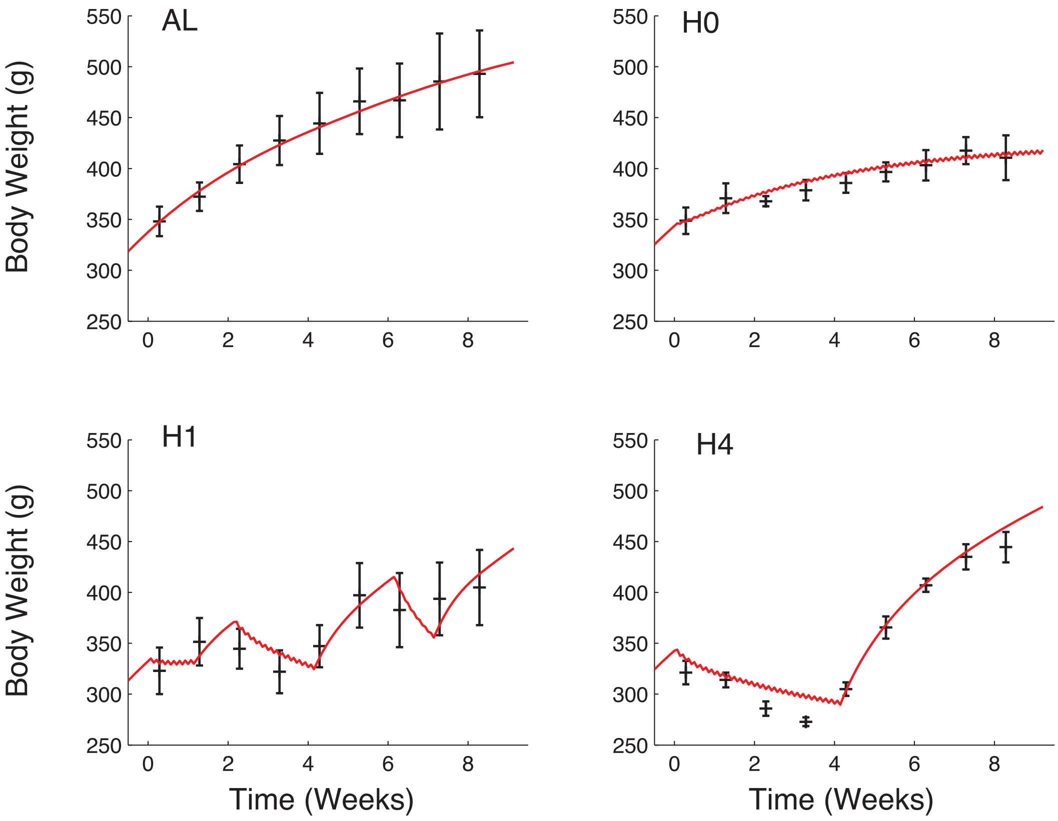

Figure 5. Simulated evolution of body weight (red line) compared to experimental data (mean ± standard deviation in black). In each group, the food input matches the experimental patterns and the first 5 days of the simulation were conducted with *Ad libitum* diet to be closer to the experiment. Parameter values were estimated with data from groups AL and H1 and predictions were made with these parameter values on groups H0 and H4. Top left: AL; top right: H0; bottom left: H1; bottom right: H4.
doi:10.1371/journal.pone.0100073.g005

equation (8). Initial condition for R is chosen to be equal to R_0 determined at step 1 as initial food is Ad libitum.

Akaike Information Criteria (AIC) was computed to compare the ability of the current model and of a model without memory (using equations (1)-(7) and R constant as for the AL case) to reproduce the data. $AIC = n\ln(RSS/n) + 2k$ with n the number of points used to evaluate the results, RSS the residual sum of squares and k the number of estimated parameters.

Approximate bayesian computation (ABC) was used to calculate a distribution of the computed parameter values, starting with uniform sampling around optimized parameters. Runs with a residual sum of squares smaller than a certain level RSS_{opti} (defined using the result of the optimization process) were selected; here the threshold was equal to 1.3 RSS_{opti} (see Table 5 for means and standard deviations of these distributions).

Predictions

Following the estimation procedure (see previous paragraphs), the model was tested with the patterns of food input corresponding to the two other groups of rats (H0 and H4) to evaluate its predictive capacity. Parameter values determined for groups AL and H1 were used. As all the rats were supposed to be similar (same origin and age), we used the same parameter values for each group.

If another group (H0 or H4) was chosen at step 3 of the estimation procedure instead of H1, the set of parameters associated with R was different. However the RSS for each set of parameters were close from one another. Hence, the simulated data will be better for the chosen group than it will for the other groups. The data from each group could be fitted individually to have better results but this would suppress the predictive capacity of the model.

Statistical Analysis

All results are presented in the form: mean ± standard deviation.

Normality of the samples was tested using Shapiro-Wilks test. Statistical comparison was performed using Mann-Whitney test for two groups, and an analysis of variance (ANOVA) for more than two groups. All analyses were performed using the R software (www.R-project.org).

Results

Food Availability Modifies Body Weight Dynamics

We present in this section the results of the experiments performed on rats – see the "Materials and Methods" section for details. In addition to a control group (called AL for *Ad libitum*, $n = 6$), three groups of 6 rats had their food availability modified during the 8 week long experiment. All rats experienced *Ad Libitum* feeding conditions prior to the experiment. Fig. 1 describes the available food time course for groups H0, H1 and H4 characterised by periods of variations of 0, 1 and 4 weeks respectively. In order to ensure a controlled total food intake, these groups were hypocaloric (around 80% of AL's average intake).

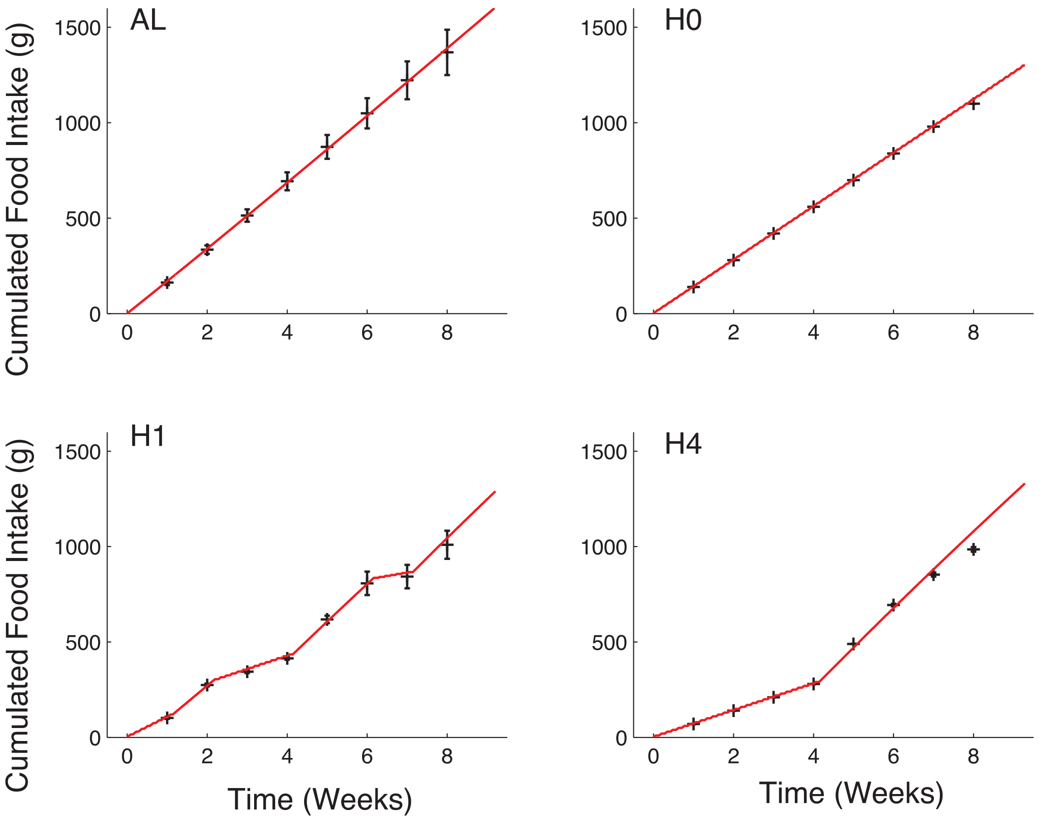

Figure 6. Evolution of cumulated food intake predicted by the model (red curve) compared to experimental data (black crosses: mean ± sd). Available food in the simulation corresponds to experimental patterns in each group, with *Ad libitum* diet in each group at the beginning. Results from groups AL and H1 correspond to the parameter estimation process while results for groups H0 and H4 correspond to predictions. Top left: AL; top right: H0; bottom left: H1; bottom right: H4.
doi:10.1371/journal.pone.0100073.g006

Group H0 was daily fed with a constant amount of food with no variations. Group H1 was daily fed with random and uncorrelated amounts of food around the average. The feeding pattern of group H4 basically corresponds to a fasting experiment for 4 weeks (less than 60% of the AL's average intake) followed by a refeeding in the following month. In the three hypocaloric groups, the total amount of food provided to each rat during the whole experiment was the same (1120 g in 8 weeks corresponding to 14.07 MJ – see Fig. 1).

At the end of the experiment, individuals were sacrificed and fat mass, muscles masses and some organ masses were collected and weighted. Table 1 displays the values along with an initial control group sacrificed on the first day of the experiment (called D0 for "day 0"). As expected, body weight is smaller for the groups with reduced food (H0, H1 and H4) compared to group AL ($p=0.00086$) and different between the 4 groups ($p=0.0008$ for the ANOVA). There is no significant evidence that distributions of body weights in each group are not normal ($p-values$ between 0.21 and 0.97).

As shown on Fig. 4 A, a difference in final body weight exists within hypocaloric groups H0, H1 and H4, the corresponding $p-value$ is slightly above the 5% threshold ($p-value=0.0636$). Pairwise comparison yields significant differences between H0 and H4 ($p-value=0.02056$) whereas total food consumption (see Fig. 4 B) is significantly different between the two groups ($p-value=0.005$), in the *opposite direction*. Rats in group H4 have a higher body weight although they ate less food than rats from group H0. No such differences are observed for group H1.

These results suggest that an energy expenditure adaptation occurs according to the amount of food consumed. This is summarized on Fig. 4 C which shows the variations of body weight during the experiment as a function of the total amount of food

Table 6. Akaike Information Criteria.

	H0	H4	H1
AIC with memory	375	368	445
AIC without memory	382	413	448

AIC (Akaike Information Criteria) for groups H0, H4 and H1 to compare results of the model with and without memory. $AIC=n\ln(RSS/n)+2k$ with n the number of points used to evaluate the results, RSS the residual sum of square and k the number of parameters estimated. AIC is smaller in the model with memory, even if there are more parameters: this model is more adapted to explain these data.
doi:10.1371/journal.pone.0100073.t006

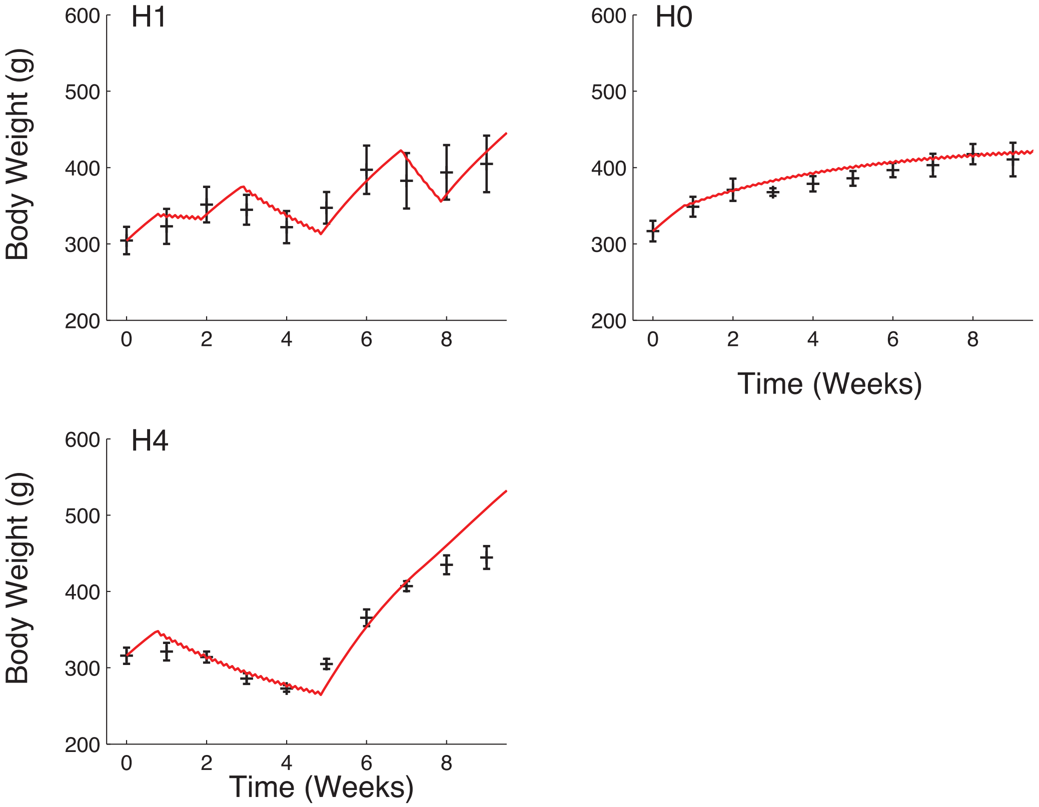

Figure 7. Predicted body weight with a constant rate of energy expenditure R compared to experimental results. Simulation for group H1 corresponds to the parameter estimation without memory (estimation of R_0 and other parameter values obtained for group AL). Predicted body weight in this case does not match experimental results. In particular, body weight is slightly overestimated for group H0 while in cases H1 and H4, the amplitude of variations is too important due to the absence of adaptation to food intake.
doi:10.1371/journal.pone.0100073.g007

consumed. All data points are plotted and the convex hull has been coloured according to each group. Data for group AL closely follows a linear pattern with slope 0.3 which indicates that each gram of food consumed turns into 0.3 grams of body weight. The other groups do not follow the same pattern. Strikingly group H4 is well above the line indicating that its individuals ate less food but that a bigger fraction of it turned into body weight. The H1 pattern is somewhat similar but less significantly.

Body weight evolution is displayed on Fig. 2 A and is consistent with the food intake in Fig. 2 B albeit with a delay, as the increase or decrease is associated with the food intake in the previous week. As observed in previous studies, when presented with various amounts of food, rats adapt their eating pattern depending on past eating behavior. Our main experimental result is that rats adapt their energy expenditure by taking efficiently advantage of the available food when in fasting conditions and using more energy when overfed. This behavior results in different body weights for the same caloric intakes.

Mathematical Model of Food Intake and Body Weight Evolution

We show in this section the predictive power of our model of feeding behavior and food intake dynamics. The model and the equations are presented in details in the "Materials and Methods" section.

Our model describes the evolution of hunger, leptin, ghrelin, plasma glucose which is correlated with insulin, energy expenditure and body weight, composed of fat and lean mass (see Table 3 for a list of the variables and their units). This model allows to describe hunger, defined as the amount of food needed by the organism, by computing the dynamics of food intake in the short term. Energy expenditure is described as a function of the rate of energy expenditure, fat-mass and fat-free mass. It includes a delay equation describing the variations of the rate of energy expenditure R. The evolution of R depends on the comparison of short-term food intake with long-term food intake (see equation (8)). Fig. 3 describes the components of the model and Table 4 describes the parameters as well as their units. The fitting procedure used to determine some parameter values is fully described in the "Materials and Methods" section. It uses only AL and H1 as training data sets.

Fig. 5.AL and 5.H1 show the results of the parameter estimation on groups AL and H1, illustrated on body weight evolution. Simulations are accurate for both groups, as expected from the parameter estimation process. In addition, the model correctly predicts the results on the validating data sets: good matches are obtained for both H0 (Fig. 5.H0) and H4 (Fig. 5.H4). Variations of predicted body weight for group H4 correlate with modifications of food availability and are close to experimental values. Small daily oscillations are observed in groups H0, H1 and H4, especially when available food is below hunger. These oscillations correspond to a daily pattern of food intake: while food is available, it is consumed, resulting in an increase in body weight, then the consumption is equal to 0 and the body weight decreases. In the case of group H4, the predicted body weight at the end of the period of restriction (week 4) is slightly higher than the actual data. As the amplitude of the restriction is important, the

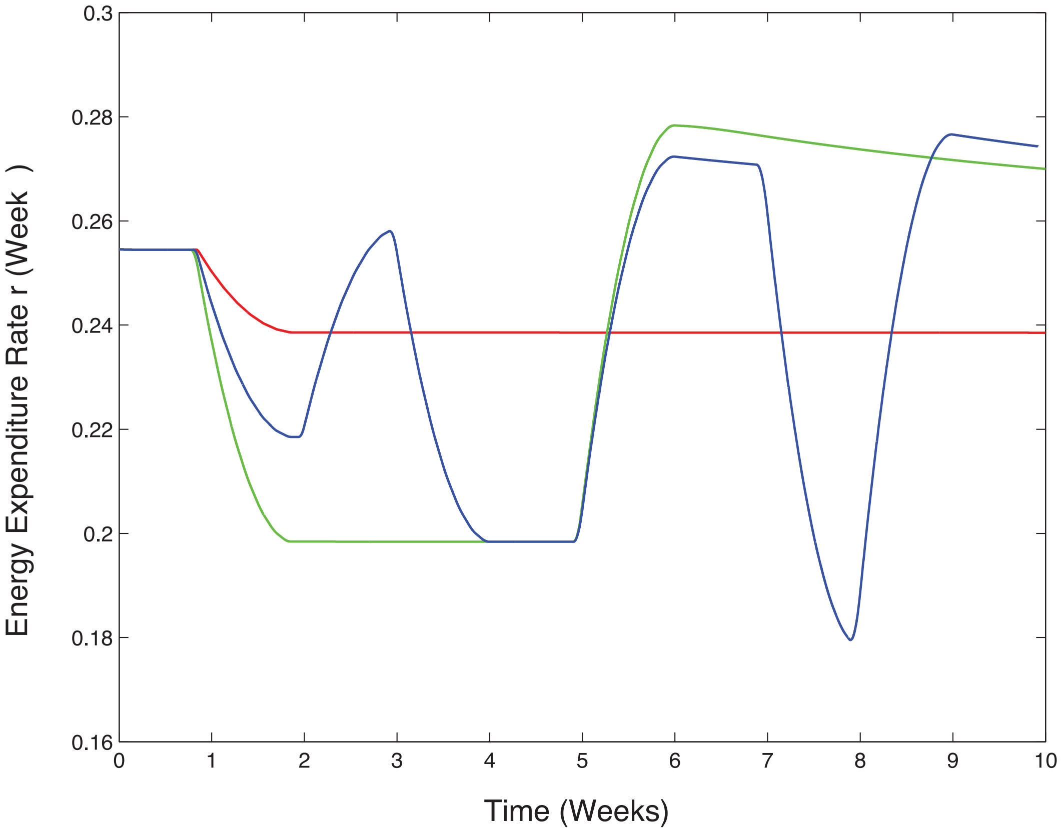

Figure 8. Predicted rate of energy expenditure variations in the cases H0 (red), H1(blue) and H4 (green), starting from the same initial condition. *R* value is stabilizing to a different value after a few days if the food pattern is followed for a long enough time (>8 days). Changes occur when the food availability is modified.
doi:10.1371/journal.pone.0100073.g008

adaptation could be less efficient in reality than it is in the model. There are also other phenomena such as environmental conditions, excluded here for simplicity, that could influence this adaptation.

Food availability is the only input of the model (see "Materials and Methods" section). It is defined according to the experimental pattern, including the five days of *Ad libitum* diet at the beginning. Our model correctly predicts food intake pattern, as shown on Fig. 6. In particular, for groups H1 and H4, the model predicts leftover food as observed in reality. In all cases, predicted food intake is a close match to the experimental data.

A Metabolic Memory is Necessary to Explain the Observed Data

The hypothesis that adaptation of the rate of energy expenditure is performed with a memory is included in equation (8) – namely the variable R is modified with a memory of the food intake in the past. As explained in the previous section, this model leads to an accurate reproduction of the experimental data. To test the relevance of this memory in the system, simulations were run with a constant value of R equal to the initial value R_0 of the rate of energy expenditure. The value of R_0 was obtained using the estimation procedure described in the section "Materials and Methods" without any memory of the past food intake and data from group H1. The model without memory was then applied to groups H0 and H4.

The values of the residual sum of squares are higher without memory than in the simulations with a non-constant R for groups H0, H1 and H4. Moreover the data are no longer well explained and predicted without memory (see Fig. 7). Akaike Information Criteria allows us to objectively compare these two different models (see Table 6). For each group, AIC is lower with memory than without, indicating that this model better explains our data despite the extra parameters to estimate. One may notice that better results could be obtained for the model without memory, by evaluating R_0 in each group separately, but the model would not be predictive anymore. For group AL, the memory did not impact the score, as expected: the rats are not submitted to caloric restrictions so they don't need to adapt their rate of energy expenditure to avoid weight variations.

Hypothesis to Explain Body Weight Differences

The main result here is derived from the evolution of the rate of energy expenditure. The variations of R are subjected to a delay equation that takes memory of past food intake into account. The model predicts the memory to be around 8 days (see Table 4).

The important weight gain in group H4 during the last 4 weeks is then explained by the lag in the refeeding period when energy expenditure is still low (see Fig. 8) while food intake is at its highest (see Fig. 2 B). Due to the delay in the adaptation of the rate of energy expenditure, the difference between energy intake and energy expenditure is maximal during this period. In the H1 case, also submitted to important caloric variations, the period of 1 week is too short to modify the rate of energy expenditure in the same way as for group H4. The adaptation is then mitigated and the observed weight gain is less important than it is for group H4.

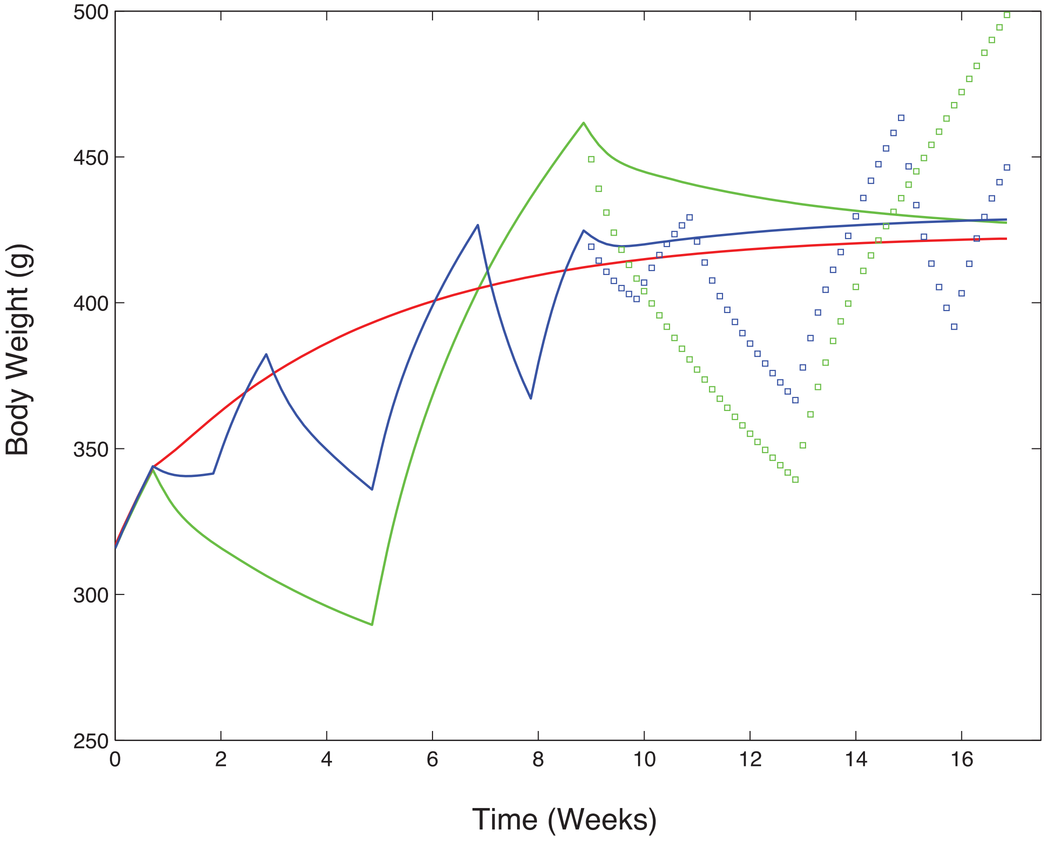

Figure 9. Predicted body weight for a 16 week experiment with different combinations of food availability patterns. In blue H1 followed by H0 (line) or H1 (squares), in green H4 followed by H0 (line) or H4 (squares) and in red twice H0. In cases with H0 in the last 8 weeks (lines), the body weight tends to the same value, whatever the past variations.
doi:10.1371/journal.pone.0100073.g009

The model was applied for 16 weeks (see Fig. 9), with H0 food pattern following H0, H1 or H4 experiment. With the same amount of food during the last 8 weeks for the three groups, the final predicted body weight tends to the same value regardless of the food pattern in the first 8 weeks. The lower food consumption for groups starting with H1 or H4 patterns does not impact this evolution. The adaptation to a constant amount of food intake leads to a fixed body weight after some time. Applying twice the same pattern (meaning the H1 diet for 16 weeks or the H4 diet for 16 weeks) leads to increases in body weights and fat mass, which reach elevated values(see Fig. 9). These variations with large amplitudes could have deleterious effects on the biological system, such as development of leptin or insulin resistances.

Discussion

In this work, we showed that food availability fluctuations can trigger body weight variations that cannot be explained by differences in the overall energy intake. In our experiment, rats submitted to the same quantity of food but distributed differently over time exhibited significant weight differences. These differences were strongest when the period of variation was high – one month of low food availability followed by one month of important food availability.

In order to explain these results, we presented a new model of body weight dynamics, describing hunger (defined as the amount of food needed by the organism), hormones and food availability dynamics. This model includes a delay equation describing variations of the rate of energy expenditure, which is adapting according to the memory of food intake. This delay equation was shown to be crucial.

After estimating the parameter values that best fit our experimental data, we showed that our model was able to both explain and predict food intake and body weight dynamics from our experimental results. We also showed that without the memory of food intake, the model cannot correctly reproduce the experimental data, which stresses that this adaptation is essential, in particular when food availability is low. Indeed, our model predicts that a period of caloric restriction leads to an increase in hunger and a decrease in the rate of energy expenditure. Ending these restrictions triggers a higher food consumption and a larger energy storage, with an increased rate of energy expenditure matching the food intake pattern. However this increase takes time to occur and during this delay period, a high amount of food is consumed while the energy expenditure remains low. We estimated a lag of 8 days which explains why quicker variations did not lead to any increase in weight. This provides a simple explanation for weight variations. A similar phenomenon is observed in humans and could explain why people submitted to very strict diets tend to gain more fat when they stop dieting, as their bodies have adapted to the reduced food consumption [25].

Although individual variability may play an important role when describing body weight variations and food intake dynamics, we did not focus on this aspect and rather considered an average behavior. The model proved its efficiency to describe the data. From the experimental results (Fig. 2), one may note that

individual variability is globally initially low and increases with the duration of the experiment. Consequently, validation of the model's predictions on the evolution of body weight during a period of time greater than 8 weeks should be supported by additional experiments and could benefit from considering variability.

The model has largely ignored some phenomena such as aging processes which affect the rate of energy expenditure, appetite or sensitivity of the system to stimuli. Indeed feeding behavior can be extremely complex especially regarding food content and palatability. Also, leptin and insulin resistances are not included in this model but are known to have an influence on the regulation of appetite and storage of fat mass following an important weight gain. Including some of these phenomena could result in a better description of the system and help enhancing our understanding of the mechanisms behind these adaptations. Nevertheless, the approach developed in this work, based on innovative mathematics and the use of a simple model, proved to be relevant to describe this physiological system.

Acknowledgments

The authors thank the reviewers for their helpful comments and Dr. David Parsons for his help in writing the manuscript.

Author Contributions

Conceived and designed the experiments: COS HAS. Performed the experiments: COS MJ. Analyzed the data: MJ. Wrote the paper: MJ FC COS HAS.

References

1. Flier JS (2004) Obesity wars: molecular progress confronts an expanding epidemic. Cell 116: 337–350.
2. Kahn SE, Hull RL, Utzschneider KM (2006) Mechanisms linking obesity to insulin resistance and type 2 diabetes. Nature 444: 840–846.
3. Abrams P, Katz LEL (2011) Metabolic effects of obesity causing disease in childhood. Curr Opin Endocrinol Diabetes Obes 18: 23–27.
4. (2000) Obesity: preventing and managing the global epidemic. report of a who consultation. World Health Organ Tech Rep Ser 894: i–xii, 1–253.
5. Barsh GS, Farooqi IS, O'Rahilly S (2000) Genetics of body-weight regulation. Nature 404: 644–651.
6. Friedman JM (2000) Obesity in the new millennium. Nature 404: 632–634.
7. Morton GJ, Cummings DE, Baskin DG, Barsh GS, Schwartz MW (2006) Central nervous system control of food intake and body weight. Nature 443: 289–295.
8. Schwartz MW, Woods SC, Porte D, Seeley RJ, Baskin DG (2000) Central nervous system control of food intake. Nature 404: 661–671.
9. Woods SC, Schwartz MW, Baskin DG, Seeley RJ (2000) Food intake and the regulation of body weight. Annu Rev Psychol 51: 255–277.
10. Crespo CS, Cachero AP, Jimnez LP, Barrios V, Ferreiro EA (2014) Peptides and food intake. Front Endocrinol (Lausanne) 5: 58.
11. Wren AM, Seal LJ, Cohen MA, Brynes AE, Frost GS, et al. (2001) Ghrelin enhances appetite and increases food intake in humans. J Clin Endocrinol Metab 86: 5992.
12. Cummings DE, Shannon MH (2003) Roles for ghrelin in the regulation of appetite and body weight. Arch Surg 138: 389–396.
13. Higgins SC, Gueorguiev M, Korbonits M (2007) Ghrelin, the peripheral hunger hormone. Ann Med 39: 116–136.
14. Duca FA, Covasa M (2012) Current and emerging concepts on the role of peripheral signals in the control of food intake and development of obesity. Br J Nutr 108: 778–793.
15. Stephens TW, Basinski M, Bristow PK, Bue-Valleskey JM, Burgett SG, et al. (1995) The role of neuropeptide y in the antiobesity action of the obese gene product. Nature 377: 530–532.
16. Covasa M (2010) Deficits in gastrointestinal responses controlling food intake and body weight. Am J Physiol Regul Integr Comp Physiol 299: R1423–R1439.
17. Duca FA, Zhong L, Covasa M (2013) Reduced cck signaling in obese-prone rats fed a high fat diet. Horm Behav 64: 812–817.
18. Beck B, Musse N, Stricker-Krongrad A (2002) Ghrelin, macronutrient intake and dietary preferences in long-evans rats. Biochem Biophys Res Commun 292: 1031–1035.
19. Friedman JM, Halaas JL (1998) Leptin and the regulation of body weight in mammals. Nature 395: 763–770.
20. Garrow JS (1987) Energy balance in man–an overview. Am J Clin Nutr 45: 1114–1119.
21. Lowell BB, Spiegelman BM (2000) Towards a molecular understanding of adaptive thermogenesis. Nature 404: 652–660.
22. Tremblay A, Royer MM, Chaput JP, Doucet E (2013) Adaptive thermogenesis can make a difference in the ability of obese individuals to lose body weight. Int J Obes (Lond) 37: 759–764.
23. Wijers SLJ, Saris WHM, van Marken Lichtenbelt WD (2009) Recent advances in adaptive thermogenesis: potential implications for the treatment of obesity. Obes Rev 10: 218–226.
24. Evans SA, Parsons AD, Overton JM (2005) Homeostatic responses to caloric restriction: influence of background metabolic rate. J Appl Physiol (1985) 99: 1336–1342.
25. Hall KD, Sacks G, Chandramohan D, Chow CC, Wang YC, et al. (2011) Quantification of the effect of energy imbalance on bodyweight. Lancet 378: 826–837.
26. de Graaf AA, Freidig AP, Roos BD, Jamshidi N, Heinemann M, et al. (2009) Nutritional systems biology modeling: from molecular mechanisms to physiology. PLoS Comput Biol 5: e1000554.
27. Tam J, Fukumura D, Jain RK (2009) A mathematical model of murine metabolic regulation by leptin: energy balance and defense of a stable body weight. Cell Metab 9: 52–63.
28. Guo J, Hall KD (2009) Estimating the continuous-time dynamics of energy and fat metabolism in mice. PLoS Comput Biol 5: e1000511.
29. Guo J, Hall KD (2011) Predicting changes of body weight, body fat, energy expenditure and metabolic fuel selection in c57bl/6 mice. PLoS One 6: e15961.
30. van Leeuwen IMM, Kelpin FDL, Kooijman SALM (2002) A mathematical model that accounts for the effects of caloric restriction on body weight and longevity. Biogerontology 3: 373–381.
31. Chow CC, Hall KD (2008) The dynamics of human body weight change. PLoS Comput Biol 4: e1000045.
32. Hall KD (2010) Predicting metabolic adaptation, body weight change, and energy intake in humans. Am J Physiol Endocrinol Metab 298: E449–E466.
33. Hall KD (2007) Body fat and fat-free mass inter-relationships: Forbes's theory revisited. Br J Nutr 97: 1059–1063.
34. Horgan GW (2011) The behaviour of a neutral model of weight regulated only by body mass. J Theor Biol 270: 1–6.
35. Hall KD (2006) Computational model of in vivo human energy metabolism during semistarvation and refeeding. Am J Physiol Endocrinol Metab 291: E23–E37.
36. Hall KD (2010) Mathematical modelling of energy expenditure during tissue deposition. Br J Nutr 104: 4–7.
37. Periwal V, Chow CC (2006) Patterns in food intake correlate with body mass index. Am J Physiol Endocrinol Metab 291: E929–E936.
38. Newby FD, DiGirolamo M, Cotsonis GA, Kutner MH (1990) Model of spontaneous obesity in aging male wistar rats. Am J Physiol 259: R1117–R1125.
39. Woods SC, Seeley RJ, Porte D, Schwartz MW (1998) Signals that regulate food intake and energy homeostasis. Science 280: 1378–1383.
40. Nelson KM, Weinsier RL, Long CL, Schutz Y (1992) Prediction of resting energy expenditure from fat-free mass and fat mass. Am J Clin Nutr 56: 848–856.
41. Cummings DE (2006) Ghrelin and the short- and long-term regulation of appetite and body weight. Physiol Behav 89: 71–84.
42. Campfield LA, Smith FJ (1990) Transient declines in blood glucose signal meal initiation. Int J Obes 14 Suppl 3: 15–31; discussion 31–4.
43. McCarter RJ, Palmer J (1992) Energy metabolism and aging: a lifelong study of fischer 344 rats. Am J Physiol 263: E448–E452.
44. Greenberg JA, Boozer CN (2000) Metabolic mass, metabolic rate, caloric restriction, and aging in male fischer 344 rats. Mech Ageing Dev 113: 37–48.
45. Nelder JA, Mead R (1965) A simplex method for function minimization. The Computer Journal 7: 308–313.
46. Vestergaard ET, Hansen TK, Gormsen LC, Jakobsen P, Moller N, et al. (2007) Constant intra- venous ghrelin infusion in healthy young men: clinical pharmacokinetics and metabolic effects. Am J Physiol Endocrinol Metab 292: E1829–E1836.
47. Cobelli C, Toffolo G, Ferrannini E (1984) A model of glucose kinetics and their control by insulin, compartmental and noncompartmental approaches. Mathematical Biosciences 72: 291–315.
48. Zeng J, Patterson BW, Klein S, Martin DR, Dagogo-Jack S, et al. (1997) Whole body leptin kinetics and renal metabolism in vivo. Am J Physiol 273: E1102–E1106.
